# Granulomatous Biliary Stricture Mimicking Malignancy Managed by Laparoscopy: A Diagnostic and Therapeutic Challenge

**DOI:** 10.7759/cureus.107237

**Published:** 2026-04-17

**Authors:** Rohit Singh, Ashok Kumar

**Affiliations:** 1 Surgical Gastroenterology, Sanjay Gandhi Postgraduate Institute of Medical Sciences, Lucknow, IND

**Keywords:** benign biliary stricture (bbs), biliary surgery, granulomatous biliary stricture, indeterminate biliary stricture, laparoscopic surgery, malignant biliary stricture, minimally invasive gallbladder surgery, minimally invasive surgery, thick walled gall bladder

## Abstract

Diagnosing biliary strictures remains challenging, as benign inflammatory conditions may closely simulate malignancy on clinical and radiological evaluation. With proper patient selection and surgical expertise, minimally invasive surgery (MIS) provides safe staging and definitive management without compromising oncologic principles for suspicious biliary strictures. We report a 30-year-old male presenting with painless progressive jaundice, weight loss, and cholestatic jaundice with imaging features suspicious for malignancy. Despite the endoscopic decompression, the cross-sectional imaging and tumor markers were inconclusive, necessitating surgical exploration. Also, as the pre-operative suspicion was of gall bladder malignancy involving the bile duct and not cholangiocarcinoma, work-up for cholangioscopic biopsy or cytology was not done, nor was any attempt made for the biopsy by the rendezvous technique. The patient underwent laparoscopic extended cholecystectomy with bile duct excision and biliary reconstruction, and histopathology revealed granulomatous cholecystitis with choledochitis without evidence of malignancy. Granulomatous biliary disease, although uncommon, frequently mimics gallbladder or biliary cancer. Post-operatively, the patient was further evaluated and found to be negative for sarcoidosis. This scientific case report underscores the importance of maintaining a high index of suspicion for benign inflammatory mimics and recognizing that histopathology remains the definitive diagnostic standard in granulomatous biliary strictures.

## Introduction

Diagnosing biliary strictures remains challenging, as benign inflammatory conditions may closely simulate malignancy on clinical and radiological evaluation [1]. Endoscopic stenting may be done in doubtful non-cancerous strictures, but misclassification may postpone the crucial cancer treatment. Conversely, opting for surgical procedures on a thick-walled gall bladder (TWGB) with biliary strictures can subject patients to unwarranted operations that carry substantial risks of complications. Surgery may serve both diagnostic and therapeutic purposes in indeterminate biliary strictures [2]. We herein describe the presentation and management of such a case.

## Case presentation

A 30-year-old male with no medical comorbidities or prior surgical history, without any environmental or occupational exposures, presented with gradually progressive jaundice of six months' duration, associated with pruritus, loss of appetite, and significant weight loss (approximately 10 kg over five months). There was no history of abdominal pain, fever, gastrointestinal bleeding, nausea, vomiting, abdominal distension, or tuberculosis. For these complaints, he was evaluated at the hospital in his locality with blood investigation suggestive of a cholestatic liver function pattern. Blood parameters are shown in Table 1.

**Table 1:** Blood parameters-pre-stenting and post-stenting (done prior to surgery). BUN: blood urea nitrogen; TLC: total leucocyte count; PLC: platelet count; SGOT: serum glutamic-oxaloacetic transaminase; SGPT: serum glutamic-pyruvic transaminase; INR: international normalized ratio; CEA: carcino-embryonic antigen; HCT: hematocrit.

Lab parameters	Pre-stenting	Post-stenting/pre-surgery
Hb (gm/dl)/HCT (%)	12.9/36.7	13.6/43.5
TLC (×1000/ul)/DLC (%)	5.8/(N50L39)	4.08/(N56L26)
PLC (×1000/cmm)	332	231
BUN (8-25 mg/dl)	-	9.1
Serum creatinine (0.5-1.6 mg/dl)	0.85	0.5
Na+ (133-146 mmol/L)/K+ (3.8-5.4 mmol/L)	-	136/4.6
Bilirubin-total (1-1.3 mg/dl)/direct (0-0.4 mg/dl)	6.54/4.95	0.66/0.17
Total protein (6-8.4 g/dL)/albumin (3.5-5.5 g/dL)	6.3/-	6.7/3.71
SGOT (5-40 u/L)/SGPT (5-40 u/L)	83/65	58/86
Alkaline phosphatase (35-150 u/L)	112	78
INR	-	1.12
Serum CEA (0-5 ng/ml)	-	2.28
Serum CA-19.9 (0-37 IU/ml)	-	17.3

**Figure 1:**
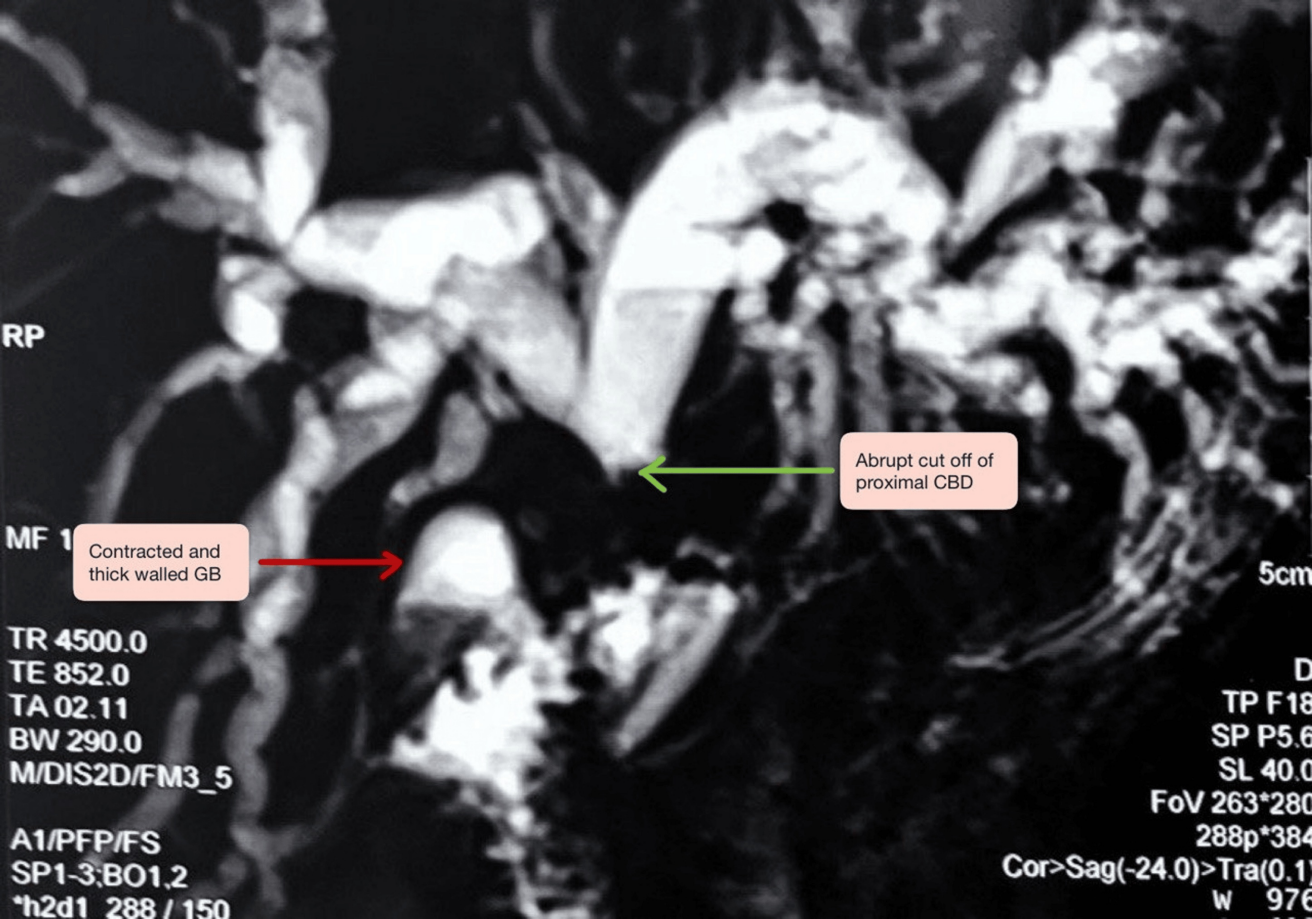
MRCP-proximal bile duct wall thickening with abrupt cut-off suspicious for a malignant biliary stricture. As the MRCP has been done prior to referring to our institute, only low-resolution films are available. Main pancreatic duct--not dilated without any pancreatic divisum. MRCP: magnetic resonance cholangiopancreatography, GB: gall bladder, CBD: common bile duct.

The patient was also evaluated with an ultrasound (USG) abdomen (September 2025-only report available), suggestive of (s/o) normal liver with intrahepatic biliary radicals' dilatation (IHBRD) with contracted and thick-walled gall bladder (TWGB) with multiple calculi with dilated common bile duct (CBD) of 11 mm. The patient was also evaluated at peripheral center with magnetic resonance cholangiopancreatography (MRCP) (September 2025) suggestive of hepatomegaly with IHBRD and an abrupt cut-off at the level of the common hepatic duct (CHD), associated with gallbladder wall thickening and proximal bile duct wall thickening with normal main pancreas with normal pancreatic duct, findings suspicious for a gall bladder malignancy involving bile duct (Figure 1).

**Figure 2:**
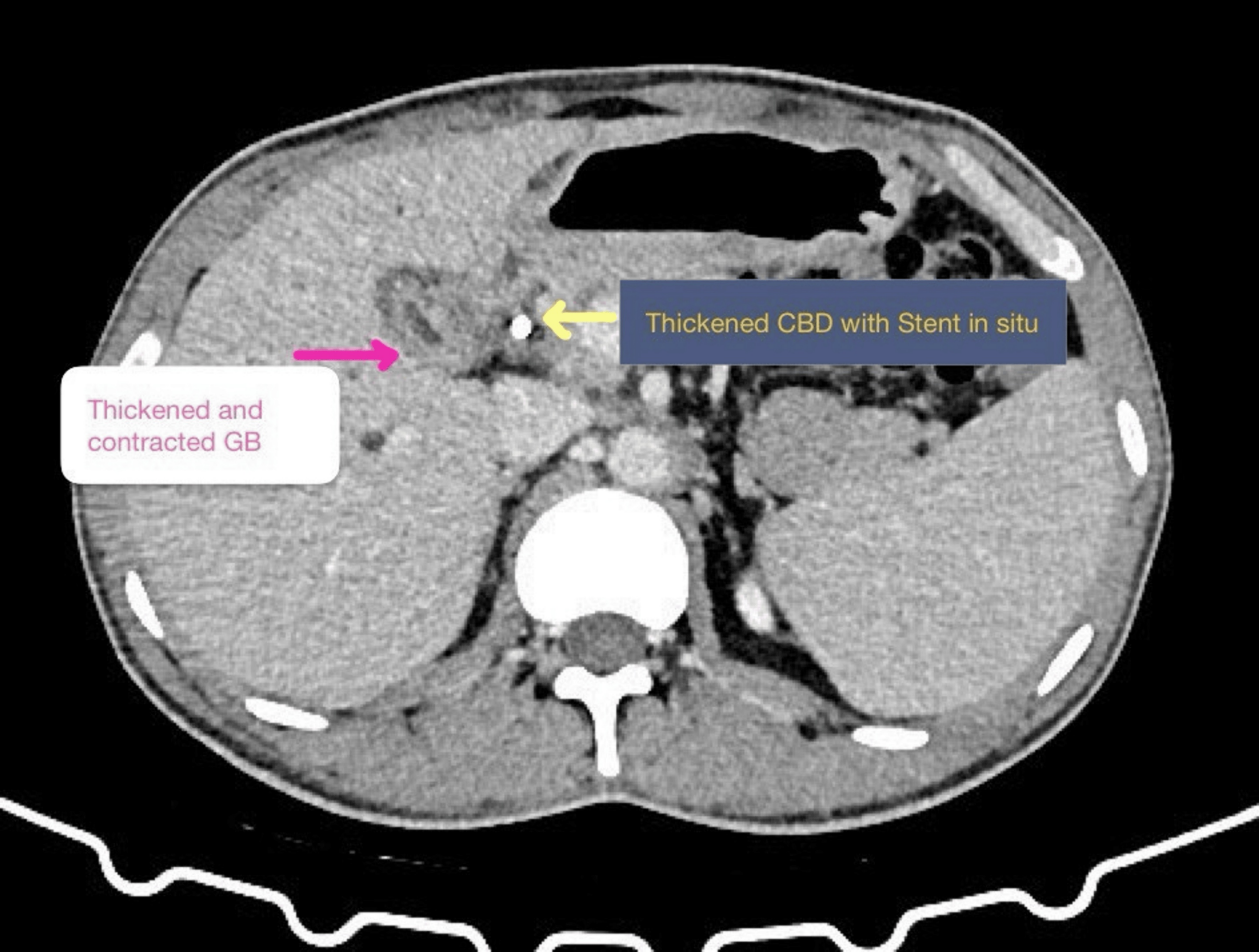
CT scan-thickened GB neck and CBD with a stent in situ. Axial section of CT scan. GB: gall bladder; CBD: common bile duct.

**Figure 3:**
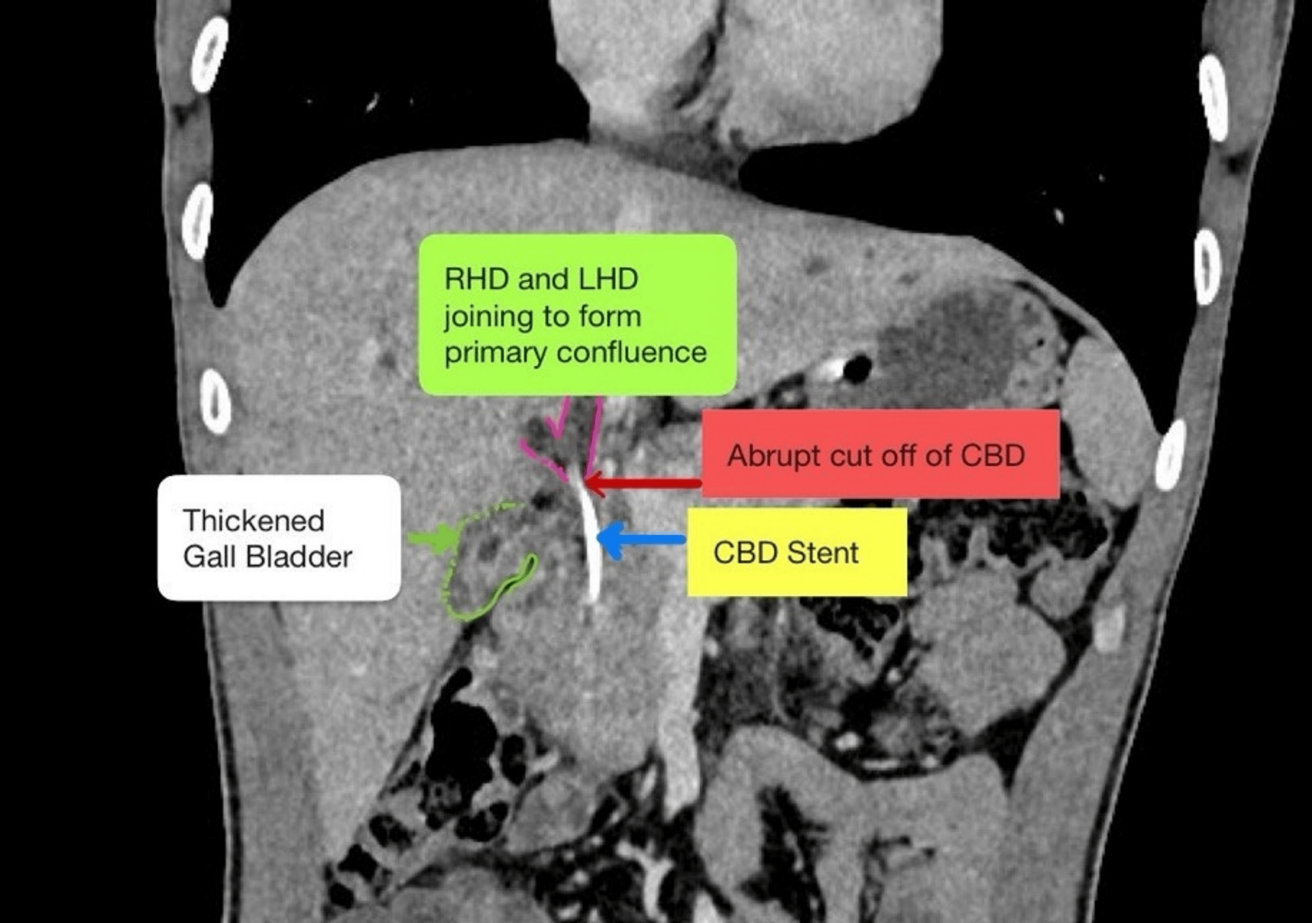
CT scan showing abrupt cut-off at proximal CBD with stent in situ and thickened GB. Coronal section of CT scan. GB: gall bladder; CBD: common bile duct; RHD: right hepatic duct; LHD: left hepatic duct.

The patient underwent endoscopic retrograde cholangiopancreatography (ERCP) (September 2025) and revealed right hepatic duct (RHD) complete cut-off, for which a 10 Fr straight stent was placed in RHD. ERCP was performed primarily for biliary decompression in view of cholestasis with pruritus, rather than for definitive diagnosis. The symptoms of jaundice and pruritus gradually improved with improvement in appetite and weight gain. The patient was then referred to our tertiary center for further management in late October 2025. On evaluation at our tertiary care center, the patient was undernourished, with a body mass index (BMI) of 18.4 kg/m² but good functional status. Vital parameters were stable, and he was afebrile. General examination revealed no abnormalities. Systemic examination was unremarkable. Evaluated with a CT scan of the abdomen suggestive of hepatomegaly (18 cm), with a thick-walled GB with a thickened cystic duct with close association with CHD near the GB neck and a thickened CBD with a CBD stent in situ, with bilobar IHBRD with bilobar pneumobilia without significant lymphadenopathy (Figures 2, 3). The pancreas and spleen were normal.

There were no clinical features suggestive of systemic IgG4-related disease, including salivary or lacrimal gland enlargement, autoimmune pancreatitis, or retroperitoneal fibrosis. Given the young age and absence of metastatic disease and TWGB with thickened bile duct, a laparoscopic approach was chosen to allow accurate staging, frozen section assessment, and definitive management. As the pre-operative suspicion was of gall bladder malignancy involving the bile duct, no further evaluation with cholangioscopic biopsy was done, and the patient was taken up for upfront surgery. The patient underwent laparoscopic extended cholecystectomy with CBD excision with Roux-en-Y hepaticojejunostomy (RYHJ) in December 2025. 

Operative findings

**Figure 4:**
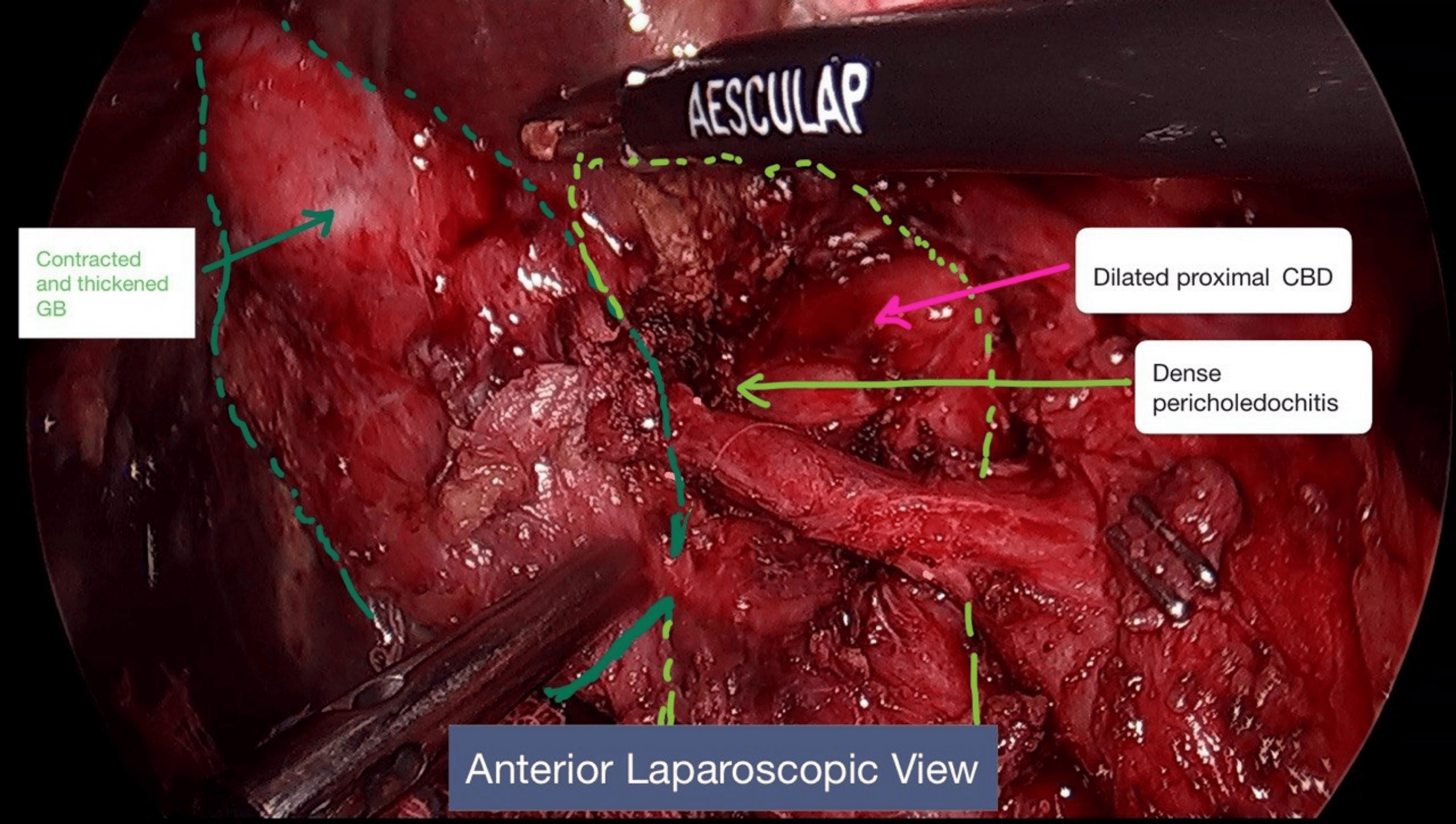
Anterior laparoscopic view-intra-operative image. Contracted and thickened GB with dense pericholedochitis and dilated proximal CBD. Calot's was frozen. The horizontal structure anterior to the CBD initially appeared to be an RHA, but on posterior dissection, it became clear that the RHA was behind the thickened posterior peritoneal fold, and the transverse structure in front was just a fibrotic band. GB: gall bladder; CBD: common bile duct; RHD: right hepatic duct; RHA: right hepatic artery.

**Figure 5:**
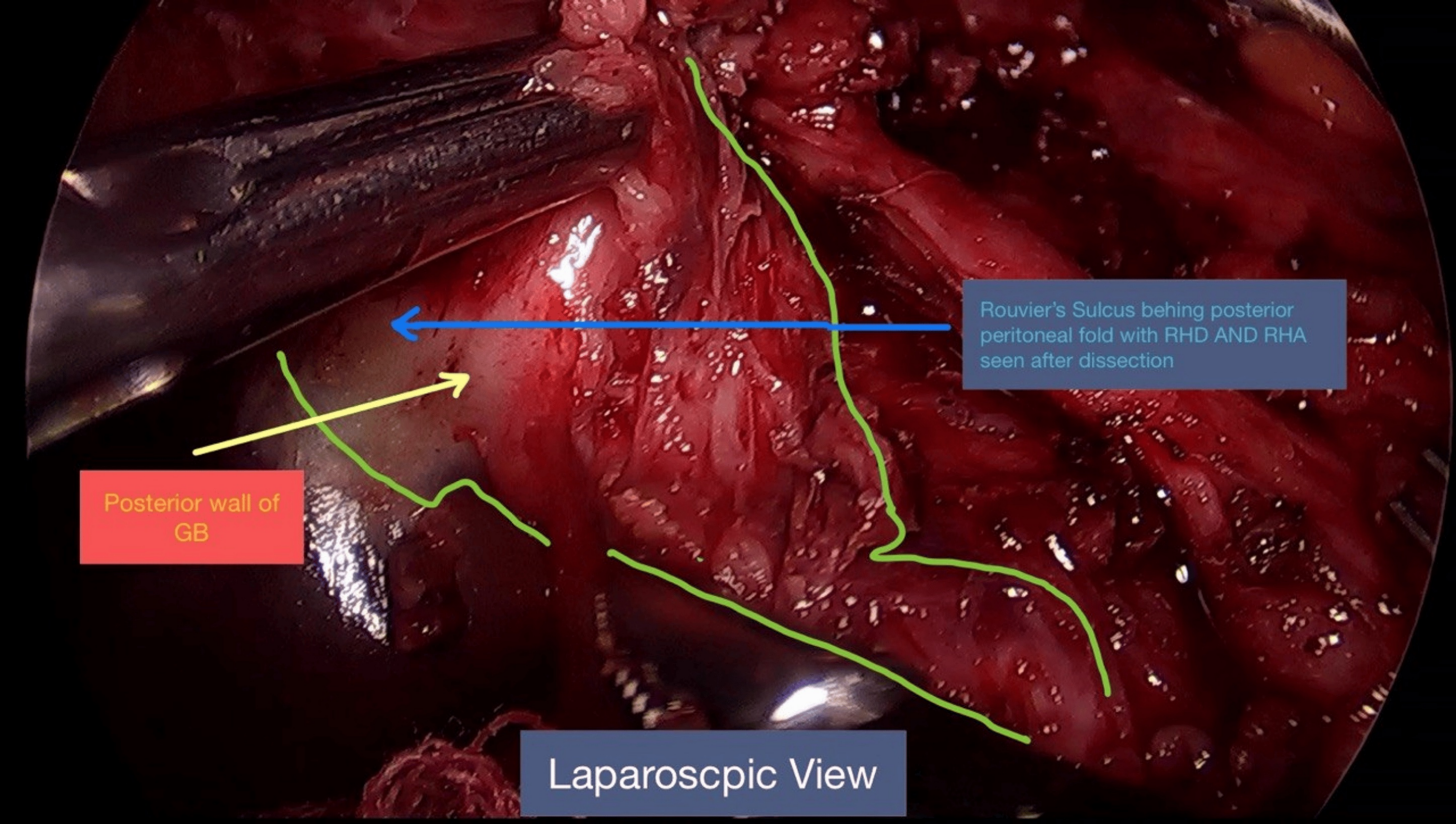
Posterior laparoscopic view. Rouvier's sulcus was hidden behind the thickened posterior peritoneal fold over the GB. GB: gall bladder; RHD: right hepatic duct; RHA: right hepatic artery.

No evidence of metastasis or ascites. The gall bladder was contracted, firm, and thickened, and its neck was adhered to the CBD (Figures 4, 5).

**Figure 6:**
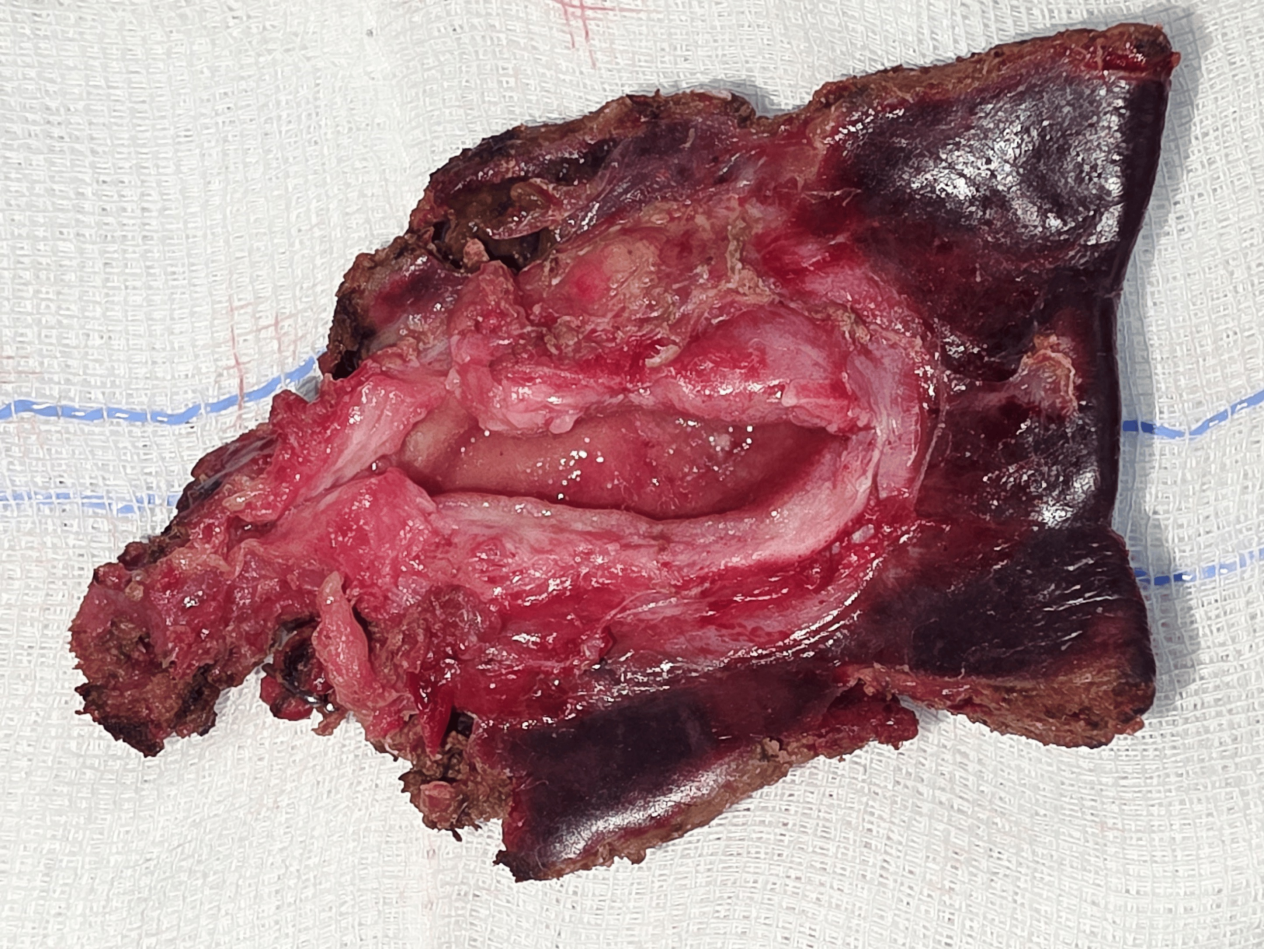
Gross cut section of GB showing thickened wall with a wedge of liver with a thickened adjoining bile duct. GB: gall bladder.

CHD was dilated, sub-hilar CHD, proximal and mid CBD was thickened, and distal CBD was normal. Choledochotomy done and single double pigtail (DPT) stent removed. Dense pericholedochitis is present. Thickened proximal portion of CBD sent for frozen--reported to be negative for malignancy (containing granuloma with epithelioid cells). A 2 cm wedge of liver was excised (till the report of frozen was validated). Proximally, CBD is divided below the hilum and distally at the supra-duodenal part of CBD, 0.5 cm distal to the thickening. The distal end of the CBD was closed. RYHJ done. Cut section-GB uniformly thick-walled, no evidence of polyp or mass (Figure 6).

**Figure 7:**
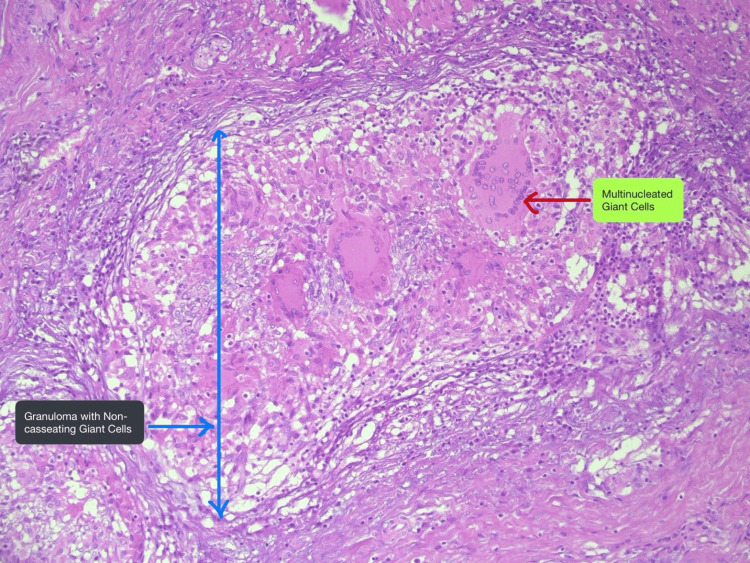
Histopathological image showing a non-caseating granuloma with a giant cell. 20× magnification of a histopathological image.

The proximal and mid portions of the CBD walls were circumferentially thickened and firm. Final histopathology of GB was granulomatous inflammation with no foam cells (negative for xanthogranulomatous lesion), with epithelioid giant cells and negative for mycobacteria on Ziehl-Neelsen (ZN) staining and CBD suggestive of no evidence of malignancy (Figure 7).

Serum angiotensin-converting enzyme (ACE) level, serum erythrocyte sedimentation rate (ESR) and fecal calprotectin was sent post-operatively which was also normal not suggestive of sarcoidosis or Crohn's disease (although there was no environmental or occupational exposures, the further work-up for tuberculosis, sarcoidosis and Crohn's disease was done to find out any pre-symptomatic disease as the histopathology was not suggestive of any xanthogranulomatous inflammation and in India the prevalence for tuberculosis is high). The post-operative hospital stay was uneventful, and the patient is doing well in follow-up without any bile leak or any features suggestive of cholangitis.

## Discussion

Benign rare causes of TWGB with biliary stricture, such as tubercular choledochitis, fungal causes, xanthogranulomatous disease, and chronic inflammatory causes, were often evident with obstructive jaundice, cholangitis, weight loss, and radiological findings indistinguishable from malignancy, including hilar strictures, “double duct” signs, lymphadenopathy, and ductal wall enhancement [3,4]. Despite advances in diagnostic modalities, including MRCP, ERCP-based tissue sampling, and tumor marker evaluation, preoperative differentiation remains challenging due to limited sensitivity and specificity, with up to 50% of cases remaining indeterminate [2,5-8]. ERCP-based brush cytology demonstrates limited sensitivity, which improves modestly with combined sampling techniques, though negative results do not reliably rule out malignancy. Intraductal ultrasonography (IDUS) provides high-resolution assessment of bile duct wall characteristics and may improve diagnostic accuracy when used adjunctively, but its utility is limited by operator dependence and lack of standardized criteria. Endoscopic ultrasound (EUS) with or without fine-needle aspiration (FNA) offers high specificity but variable sensitivity [7-9].

Historically, major deterrents to minimally invasive surgery (MIS) have been largely mitigated by advances in surgical technique, including minimal tumor handling, avoidance of bile spillage, routine use of specimen retrieval bags, and adherence to oncologic principles, with modern studies reporting no increase in port-site or locoregional recurrence as compared to open surgery [2,10]. Additionally, MIS allows accurate staging laparoscopy, preventing non-therapeutic laparotomies in patients with occult metastatic disease, and facilitates a stepwise strategy with intraoperative frozen section assessment to tailor the extent of resection [10].

If there is a pre-operative suspicion of GB malignancy with bile duct involvement, as in this case, the case can proceed for surgery, but if there is thickening of only the bile duct, it should be properly evaluated with cholangioscopic biopsy to prevent unnecessary surgery.

Surgical resection serves both diagnostic and therapeutic purposes. A minimally invasive approach in expert centers, when feasible, allows oncologically sound resection while minimizing surgical morbidity. This case demonstrates that, in carefully selected patients, laparoscopy allows safe staging, frozen section assessment, and definitive management of indeterminate biliary strictures.

Histopathology after surgery remains the definitive diagnostic modality, underscoring the limitations of current preoperative work-up and a stepwise diagnostic approach to avoid both under- and overtreatment [2].

## Conclusions

Benign granulomatous biliary strictures can closely mimic malignant biliary obstruction, making preoperative diagnosis challenging. Since this is a single case report, there is a limitation in that there is an inherent difficulty in preoperative differentiation despite modern imaging. Despite advances in imaging and endoscopic techniques, histopathological examination remains the definitive diagnostic modality. A multidisciplinary, stepwise approach is essential to optimize outcomes and avoid unnecessary radical surgery. Minimally invasive surgery, when performed in experienced centers, can provide both diagnostic certainty and definitive treatment.
